# Kinetics and Thermodynamics of DNA Processing by Wild Type DNA-Glycosylase Endo III and Its Catalytically Inactive Mutant Forms

**DOI:** 10.3390/genes9040190

**Published:** 2018-03-30

**Authors:** Olga A. Kladova, Lev N. Krasnoperov, Nikita A. Kuznetsov, Olga S. Fedorova

**Affiliations:** 1 Institute of Chemical Biology and Fundamental Medicine (ICBFM), 630090 Novosibirsk, Russia; kladova@niboch.nsc.ru; 2 Department of Natural Sciences, Novosibirsk State University, 630090 Novosibirsk, Russia; 3 New Jersey Institute of Technology, Department of Chemistry and Environment Sciences, University Heights, Newark, NJ 07102, USA; lev.n.krasnoperov@njit.edu

**Keywords:** DNA repair, endonuclease III, 5,6-dihydrouracil, stopped-flow enzyme kinetics, thermodynamics, fluorescence

## Abstract

Endonuclease III (Endo III or Nth) is one of the key enzymes responsible for initiating the base excision repair of oxidized or reduced pyrimidine bases in DNA. In this study, a thermodynamic analysis of structural rearrangements of the specific and nonspecific DNA-duplexes during their interaction with Endo III is performed based on stopped-flow kinetic data. 1,3-diaza-2-oxophenoxazine (tC^O^), a fluorescent analog of the natural nucleobase cytosine, is used to record multistep DNA binding and lesion recognition within a temperature range (5–37 °C). Standard Gibbs energy, enthalpy, and entropy of the specific steps are derived from kinetic data using Van’t Hoff plots. The data suggest that enthalpy-driven exothermic 5,6-dihydrouracil (DHU) recognition and desolvation-accompanied entropy-driven adjustment of the enzyme–substrate complex into a catalytically active state play equally important parts in the overall process. The roles of catalytically significant amino acids Lys120 and Asp138 in the DNA lesion recognition and catalysis are identified. Lys120 participates not only in the catalytic steps but also in the processes of local duplex distortion, whereas substitution Asp138Ala leads to a complete loss of the ability of Endo III to distort a DNA double chain during enzyme–DNA complex formation.

## 1. Introduction

Cellular DNA is constantly exposed to various exogenous and endogenous factors, among them reactive oxygen species, alkylating compounds, and ultraviolet and ionizing irradiation lead to chemical modification of nucleotides [1,2,3,4]. Recognition and excision of nonbulky DNA base lesions proceeds via the base excision repair pathway (BER), which is initiated by DNA glycosylases [5].

The enzyme endonuclease III (Endo III or Nth) is one of eight DNA glycosylases in prokaryotes [6] and recognizes a broad range of oxidized or reduced pyrimidines to initiate BER [7,8,9,10,11,12,13]. Endo III is a bifunctional DNA glycosylase and catalyzes both hydrolysis of the *N*-glycosidic bond and the β-elimination reaction of the 3′-phosphate group [14,15,16].

**Figure 1:**
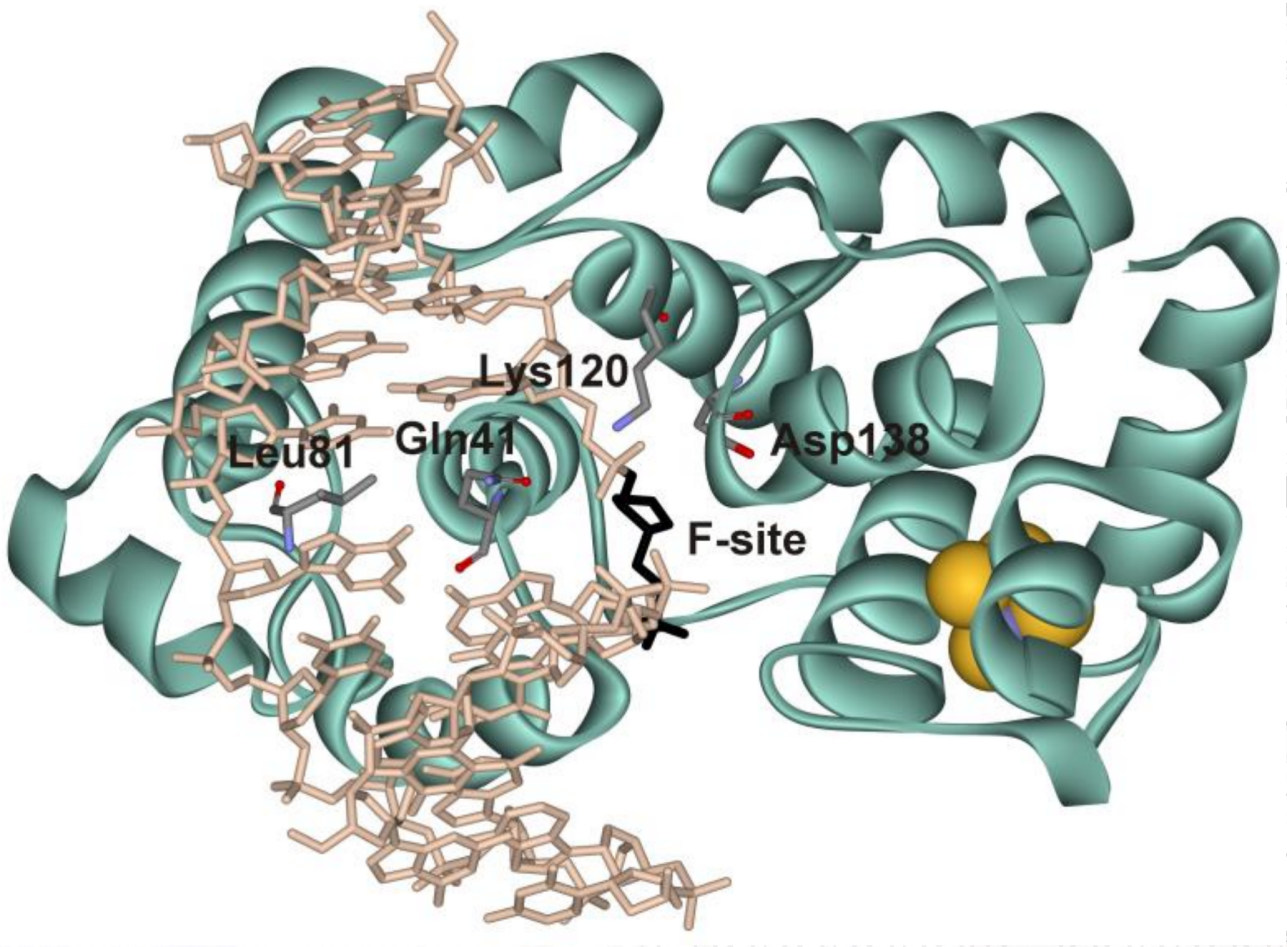
The structures of Endonuclease III (Endo III) from *Geobacillus stearothermophilus* complexed with DNA containing an F abasic site analog (PDB ID 1P59). The F nucleotide (black) everted into the active site of the enzyme. The catalytic Lys120 and Asp138 and void-filling Gln41 and Leu81 amino acid residues are labeled (numbers are given for the *Escherichia coli* protein).

Crystal structures of the free enzyme from *Escherichia coli* and complexes of Endo III from *Geobacillus stearothermophilus* with DNA [17,18] have revealed conformational changes both in the protein and DNA (Figure 1). Endo III induces gross conformational changes in DNA via double-chain bending approximately by 55° at the site of a lesion and pushing out opposite base from the DNA chain. The damaged nucleotide flips out from the helix and gets inserted into the active site of the enzyme. The process of formation of the enzyme–DNA catalytic complex is accompanied by the insertion of amino acid residues Gln41 and Leu81 (numbers are given for the *E. coli* protein) into the DNA void formed after the damage eversion (Figure 1).

The overall mode of interaction of Endo III with DNA is very similar to that known for other members of the helix-hairpin-helix (HhH) structural superfamily, making it possible to compare the conformational features of DNA binding and damage recognition with other well-studied members of this superfamily [16]. Pre-steady-state kinetic analysis of conformational transitions in DNA during formation of the complex with Endo III [19] has revealed that the initial step of lesion recognition most likely begins with insertion of the wedge amino acid residue Leu81 into the DNA helix. Insertion of other amino acids, eversion of the damaged nucleotide into the active site, and final adjustment of the active site to a catalytically competent state proceed at the subsequent steps of specific lesion recognition. Moreover, kinetic studies of other members of the HhH superfamily—human 8-oxoguanine-DNA glycosylase hOGG1 [20], adenine-DNA glycosylase MutY [21], and human methyl-CpG-binding enzyme MBD4 [22]—have revealed that all these enzymes follow a multistep mechanism of lesion recognition that includes step-by-step involvement of different amino acids, which, possibly, play similar roles in DNA wedging and destabilization of the damaged region. Recent studies [23,24,25,26,27,28,29] of the DNA lesion search mechanism revealed that some other DNA glycosylases use a wedge amino acid to sense damaged DNA bases before their eversion into the enzyme’s active site.

A thermodynamic analysis of catalytic-complex formation during interaction of hOGG1 [30] as well as formamidopyrimidine-DNA glycosylase Fpg [31] with human apurinic/apyrimidinic (AP) endonuclease APE1 [32] suggests that the DNA substrate binding and recognition is mainly governed by a positive entropy change due to insertion of the wedge amino acid residues into the duplex, resulting in the removal of crystalline water molecules from DNA grooves and desolvation of polar groups at the interacting interfaces; these events are required for formation of direct contacts between the enzyme and DNA [33].

Formation of the catalytic complex between Endo III and damaged DNA is accompanied by *N*-glycosidic bond cleavage and the β-elimination reaction of the 3′-phosphate group. It is assumed that hydrolysis of the *N*-glycosidic bond is catalyzed by a water molecule [34,35,36]. It is worth noting that the presence of AP-lyase activity in all bifunctional members of the HhH structural family is due to the Lys residue in the active site of these enzymes. Moreover, it has been shown that the site-directed insertion of a Lys residue into the active site of monofunctional adenine-DNA glycosylase MutY converts this enzyme into a bifunctional glycosylase [37]. Designing separation-of-function mutants has allowed researchers to identify the invariant Asp residue as the catalytic residue [38]. The kinetic data [24] also support the idea that bifunctional enzymes of the HhH structural family possess uncoupled catalytic activity [38], where Asp is responsible for hydrolysis of the *N*-glycosidic bond, while Lys is a key amino acid for the sugar-phosphate bond cleavage (β-elimination reaction).

The thermodynamic parameters of specific steps obtained in the temperature-dependent assay proved to be helpful for reliable identification of specific steps in the overall repair process based on the information that has not been available before in single-temperature studies. Therefore, in the present work, with the aim to gain a deeper insight into the mechanism of the search for damage, we performed thermodynamic analysis of the interaction of *E. coli* Endo III with specific DNA containing 5,6-dihydrouracil (DHU) and (3-hydroxytetrahydrofuran-2-yl)methyl phosphate (F-site) and nonspecific undamaged DNA based on the stopped-flow kinetic data. The multistep kinetic mechanism, the corresponding rate constants of the forward and reverse reactions, and the resultant equilibrium constants were derived here by means of temporal fluorescence traces of a fluorescent analog of a natural base, 1,3-diaza-2-oxophenoxazine (tC^O^), introduced into the DNA substrates, across very diverse (five orders of magnitude, from milliseconds to a hundred seconds) timescales and extended temperature ranges. 1,3-Diaza-2-oxophenoxazine, a fluorescent analog of the nucleoside cytidine, retains its Watson–Crick base-pairing capacity with G, and this property is only moderately affected by temperature [39], making it a versatile probe for fluorescent measurements of the kinetics at different temperatures.

To determine the function of catalytic amino acid residues Lys120 and Asp138 of the enzyme in DNA-binding processes, recognition of damage, and the catalyzed reaction, mutant forms of the enzyme, K120A and D138A, were constructed.

These experiments allowed us: (i) to observe conformational changes of the substrates in real time during a lesion search, damaged-base recognition, and catalysis; (ii) to identify the role of catalytically important amino acids during the enzymatic pathway; and (iii) to calculate thermodynamics of the fast steps of DNA lesion recognition and processing, and improve our understanding of the molecular mechanism of lesion recognition by Endo III.

## 2. Materials and Methods

### 2.1. Construction of the pET28c-Endo III Expression Vector and Mutagenesis

The PCR amplicon (DNA fragment) that contained the Endo III gene and *Nde*I and *Bam*HI restriction sites was prepared from plasmid pNth10 [13,19,40] and specific primers 5′-GGAATTCCATATGAATAAAGCAAAACGCC-3′ and 5′-GCGGATCCTCAGATGTCAACTTTCTCTTTG-3′. Plasmid pNth10 containing the Endo III gene was kindly provided by M.K. Saparbaev (Groupe Réparation de l'ADN, Université Paris-Sud XI, Institute Gustave Roussy, France) [40]. The size of the DNA fragment was 654 bp. The PCR products were separated on 1.0% agarose/TAE gels, excised, and purified with the QIAquick PCR purification kit (Qiagen, Hilden, Germany). Finally, the gene of Endo III was cloned into the pET28c expression vector via *Nde*I and *Bam*HI restriction sites and sequenced.

Mutations Lys120Ala and Asp138Ala within the Endo III coding sequence were generated by means of a site-directed mutagenesis kit (QuikChange XL, Stratagene La Jolla, CA, USA).

### 2.2. Protein Expression and Purification

Wild type (WT) Endo III and its mutant forms were purified from *E. coli* Rosetta 2 cells transformed with plasmid pET28c-Endo III. Cells of *E. coli* Rosetta 2 were grown in the Luria–Bertani (LB) medium (1 L) containing 50 µg/mL kanamycin at 37 °С to optical density of 0.6–0.7 at 600 nm. Then, the temperature was lowered to 20 °С, and transcription was induced by addition of isopropyl-β-d-thiogalactopyranoside to 0.2 mM. After that, the cells were incubated for 16 h and then centrifuged (12,000 rpm, 10 min). A cell suspension was prepared in 30 mL of buffer I (20 mM HEPES-NaOH, pH 7.8) containing 40 mM NaCl and a protease inhibitor cocktail (Complete, Mannheim, Germany). The cells were lysed using a French Pressure Cell Press (Thermo Electron Corporation, Needham Heights, MA, USA). All subsequent procedures were conducted at 4 °С. The cell lysate was centrifuged (30,000 rpm, 40 min), and the supernatant was adjusted to 200 mM NaCl and loaded onto column I (Q-Sepharose Fast Flow, Amersham Biosciences, Uppsala, Sweden) with subsequent washing with buffer solution I (20 mM HEPES-NaOH, pH 7.8) containing 200 mM NaCl. Fractions containing the protein were collected and loaded onto column II (HiTrap-Chelating™, Amersham Biosciences) in buffer solution II (20 mM HEPES-NaOH, pH 7.8) containing 500 mM NaCl and 20 mM imidazole. Chromatography was performed in buffer solution II and a linear gradient of 20 → 500 mM imidazole. The solution’s absorbance was detected at a wavelength of 280 nm. The protein purity was determined by gel electrophoresis. Fractions containing the Endo III protein were dialyzed against a buffer (20 mM HEPES-NaOH, pH 7.5, 1 mM EDTA, 1 mM dithiothreitol, 250 mM NaCl, 50% glycerol) and stored at −20 °C.

### 2.3. Oligodeoxynucleotides

The sequences of oligodeoxyribonucleotides used in this work are listed in Table 1. The oligodeoxyribonucleotides were synthesized by the standard phosphoramidite method on an ASM-700 synthesizer (BIOSSET, Novosibirsk, Russia) in the Laboratory of Biomedical Chemistry, of Institute of Chemical Biology and Fundamental Medicine of Siberian Branch of Russian Academy of Sciences. Phosphoramidites, including tC^O^ and DHU-containing monomers, were purchased from Glen Research (Sterling, VA, USA). Duplexes were prepared by annealing the modified and complementary strand at a 1:1 molar ratio.

DNA duplexes contained a centrally placed specific DHU lesion, an uncleavable analog of an AP-site (3-hydroxytetrahydrofuran-2-yl) methyl phosphate (F-site), or an undamaged nucleotide (Table 1). The DHU-containing substrate (DHU-substrate) was subject to the full enzymatic cycle, which includes DNA binding, *N*-glycosidic-bond cleavage, β-elimination, and a product release. The undamaged DNA duplex (G-ligand) and the duplex containing an F-site (F-ligand) revealed conformational changes in DNA during the binding with Endo III uncomplicated by catalytic steps.

### 2.4. Stopped-Flow Experiments

These experiments were conducted essentially as described previously [41,42,43]. A SX.18MV stopped-flow spectrometer (Applied Photophysics, Leatherhead, UK) fitted with a 150 W Xe arc lamp and 2 mm path length optical cell was employed. The dead time of the instrument was 1.4 ms. The excitation wavelength was 360 nm for the tC^O^ fluorescent dye. Emission was monitored using a long-pass wavelength filter at 395 nm (Corion, Franklin, MA, USA). Endo III was placed in one instrument’s syringe and rapidly mixed with the substrate in another syringe. The concentration of substrates in all the experiments was 1.0 μM, and the concentration of Endo III was varied in the 0.5–4.0 μM range. The reported concentrations of reactants are those in the reaction chamber after mixing. Typically, each trace shown in the figures is the average of four or more fluorescence traces recorded in individual experiments. In the figures, if necessary for better presentation, the curves were manually moved apart. This procedure does not affect the results of fitting because the background fluorescence is fitted separately for each curve. All the experiments were carried out in a buffer consisting of 50 mM Tris-HCl, pH 7.5, 50 mM KCl, 1 mM EDTA, 1 mM DTT, and 7% glycerol (*v/v*) at different temperatures across the range of 5–37 °C.

### 2.5. Product Analysis

To analyze the products formed by Endo III, the substrates were 5′-^32^P-labeled with phage T4 polynucleotide kinase and γ-^32^P-ATP; the reaction was carried out under the conditions described above. The products were precipitated by adding 10 volumes of 2% LiClO_4_ in acetone. The precipitates were washed three times with 100 μL of acetone, dried, dissolved in 4 μL of water and 3 μL of loading buffer (7 M urea, 0.1% bromophenol blue, and 0.1% xylene cyanol), and analyzed by denaturing polyacrylamide gel electrophoresis (PAGE) in a 20% gel. The gels were visualized using Agfa CP-BU X-ray film (Agfa-Geavert, Mortsel, Belgium), and the autoradiograms were scanned and quantified in the Gel-Pro Analyzer software (Media Cybernetics, Rockville, MD, USA).

### 2.6. Kinetic Data Analysis

The kinetic parameters were determined by global nonlinear fitting in the DynaFit software (BioKin, Pullman, WA, USA) [44] as described previously [31,45]. The software performs numerical integration of a system of ordinary differential equations with subsequent nonlinear least-squares regression analysis. The kinetic mechanism, all relevant rate constants for the forward and reverse reactions, and the specific molar responses for all intermediate complexes were fitted.

The data were fit numerically to several kinetic schemes with *n* reversible steps followed by *m* irreversible steps and a reversible product release step. The residuals were examined, and the minimal kinetic schemes were selected based on the screen test, as done previously [45].

### 2.7. Thermodynamic Analysis

By means of the measured rate constants, the equilibrium constants *K*_i_ (*k*_i_/*k*_−i_, where *i* is the step number) were determined for a DNA substrate and ligand. The standard thermodynamic functions of the *i*^th^ equilibrium step were determined using the Van’t Hoff equation, which represents the relation between the true thermodynamic equilibrium constant (*K*_i_) and standard Gibbs energy (Δ*G*_i_^o^), the standard enthalpy (Δ*H*_i_^o^), and the standard entropy (Δ*S*_i_^o^) of the *i*^th^ reaction step [46]:

ln(*K*_i_) = −Δ*G*_i_^o^/*RT* = −Δ*H*_i_^o^/*RT* + Δ*S*_i_^o^/R (1)

The typical dependence of ln(*K*_i_) on 1/T was linear, as expected for the relatively narrow temperature range of the study. The Gibbs free energies Δ*G*^o^_i_ at 25 ^°^C were calculated from ln(*K*_i_) = −Δ*G*^o^_i_/RT. The validity of this approach was discussed elsewhere (e.g., [47]).

Analysis of the temperature dependence of the rate constant of chemical reaction *K*_i_ enables determination of standard enthalpy of activation (Δ*H*^o,‡^) and standard entropy of activation (Δ*S*^o,‡^) based on the transition state theory (Eyring equation [46]). For unimolecular reactions, such as the catalytic step in the reaction mechanism,

ln(*k*/T) = ln(*k*_B_/*h*) *+* (Δ*S*^o,‡^/R) − (Δ*H*^o,‡^/*RT*), (2)

where *k*_B_ and *h* are Boltzmann’s and Planck’s constants, respectively, *R* is the gas constant, *T* is absolute temperature in Kelvins, and *k_i_* denotes the rate constant of chemical step *i*.

## 3. Results

### 3.1. Rationale

Recently, we presented a kinetic study of conformational changes in DNA during DNA binding, damage recognition, and catalysis by *E. coli* Endo III [19]. In the previous study [19], two fluorescent reporters, 2-aminopurine (aPu) placed 3′ to the damaged nucleotide and tC^O^ placed opposite to the lesion, were chosen for detection of DNA dynamics. The transient changes in the aPu and tC^O^ fluorescence during the interaction of Endo III with DNA were examined via the stopped-flow approach under conditions close to single-turnover, indicating conformational transitions in the DNA molecules. The data showed that DNA undergoes multiple conformational transformations during the formation of an enzyme–substrate complex. The initial step of the kinetic mechanism is characterized by destabilization of the double helix (which can occur due to local melting, bending of the duplex, or insertion of the Leu81 residue) and by attempts of the enzyme to flip out the damaged nucleotide from the duplex. It was likely that the second binding step accompanied by the incorporation of the Gln41 amino acid residue into a double helix and simultaneous adjustment of the active site to achieve the catalytically competent state.

In contrast to our previous study [19], where 12-nucleotide duplexes as DNA substrates were studied, the longer DNA (Table 1) allows us to analyze the process across a wide temperature range (5–37 °C) and to determine the thermodynamic parameters (standard Gibbs energy, standard enthalpy, and standard entropy) for each step of conformational rearrangements associated with specific recognition of a damaged DNA site and the catalytic reaction. Additionally, single mutants of Endo III were examined to identify the function of catalytic amino acid residues Lys120 and Asp138 during DNA binding and catalysis. A comparison of fluorescence kinetic data with available structures for Endo III allowed us to improve the understanding of the mechanism of Endo III interaction with specific and nonspecific sites.

### 3.2. Interactions of Endonuclease III with G-Ligand

**Figure 2:**
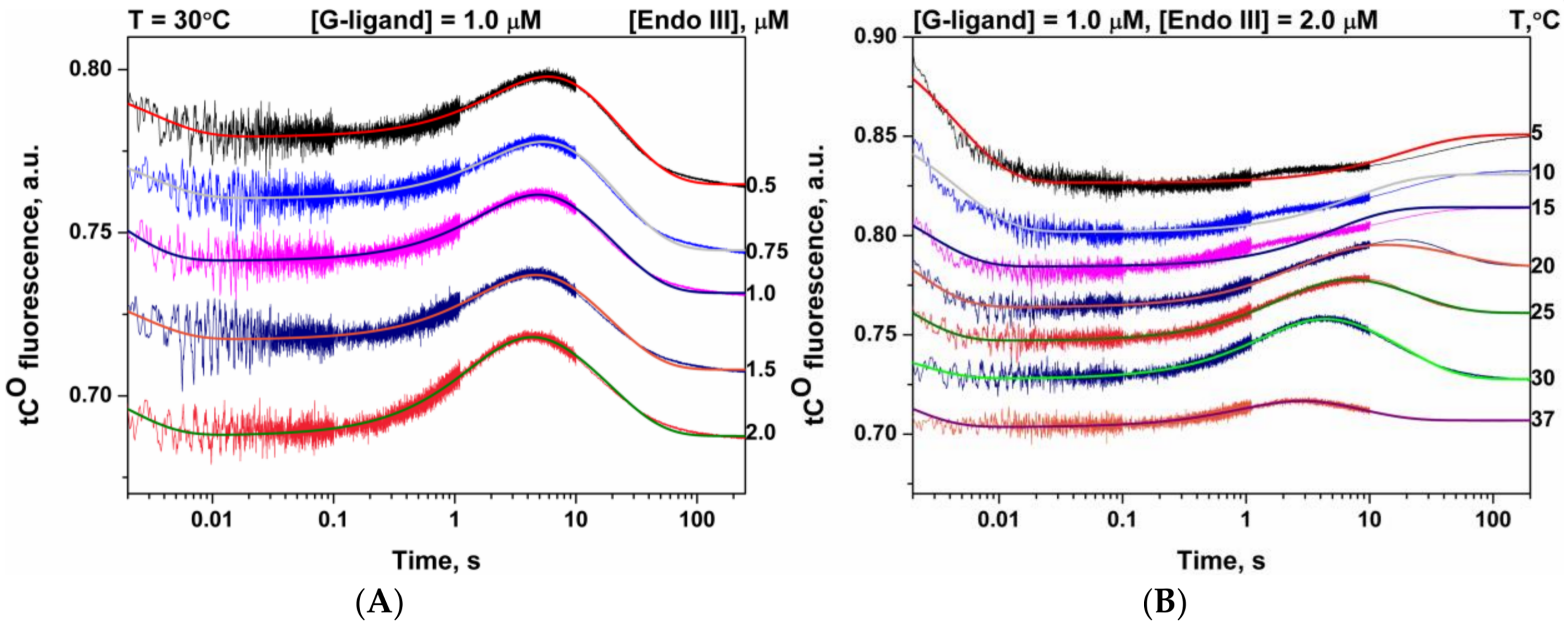
(**A**) Changes in 1,3-diaza-2-oxophenoxazine (tC^O^) fluorescence intensity during the interaction of Endo III with G-ligand (1 µM) at various concentrations of the enzyme at 30 °C. Solid curves represent the curves fitted by means of Scheme 1; (**B**) Changes in tC^O^ fluorescence intensity during the interaction of Endo III (2 µM) with G-ligand (1 µM) at different temperatures.

For nonspecific G-ligand, the set of tC^O^ fluorescence traces obtained at different concentrations of Endo III and T = 30 °C are presented in Figure 2A. Changes in tC^O^ fluorescence intensity during interaction of Endo III (2 µM) with G-ligand (1 µM) at different temperatures are shown in Figure 2B. The obtained data indicate that, in the time interval 2 ms to 200 s, the nonspecific binding of G-ligand by Endo III led to a three-phase change in tC^O^ fluorescence intensity. The rates of all phases increased with the temperature.

The first phase represents a decrease in the signal up to time points 10–100 ms. According to [48], the decrease in tC^O^ fluorescence intensity represents the transfer of this label into a more polar environment. The second phase when tC^O^ fluorescence intensity is increased proceeds up to ≈3 s (37 °C) or ≈200 s (10 °C). The third phase with the decrease of tC^O^ fluorescence intensity is observed only for T = 20–37 °C in the time range 5–200 s. Presumably, in the first phase, when enzyme forms initial contacts with one of the DNA chains, tC^O^ is detached from the complementary chain and becomes more accessible for polar solution molecules. In the second phase, the enzyme forms specific contacts with both chains of DNA. As suggested previously [19], for the DNA binding with Endo III, the side chain of Leu81 of Endo III can be partially inserted into the DNA helix during complex formation, and the side chain of Gln41 contacts the base opposite to the lesion site. Therefore, due to these interactions, the tC^O^ environment becomes less polar. During the third phase, tC^O^ fluorescence intensity decreases again. Probably, the insertion of amino acid residues into the double helix of DNA promotes the extrusion of nucleobases, including tC^O^, into the external polar side of the DNA chain.

**Scheme 1:**
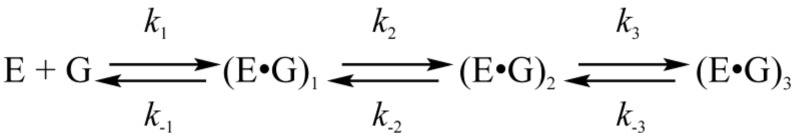
E represents Endo III, G denotes G-ligand, (E•G)_i_ are complexes of the enzyme with G-ligand, and *k*_i_ and *k*_−i_ are the individual rate constants.

In the DynaFit software [44], it was determined that the experimental curves obtained for a set of Endo III concentrations and fixed DNA concentration at each temperature fit best a kinetic mechanism containing three reversible steps, as shown in Scheme 1. The complexes (E•G)_i_ in Scheme 1 correspond to different forms of G-ligand complexed with the enzyme detected on fluorescence traces. The forward and reverse rate constants calculated by fitting of the experimental data to this mechanism are listed in Table 2. For each temperature, the equilibrium constants of individual steps *K*_i_ and association constant *K*_ass_, which represents the total DNA binding ability of enzyme, were calculated from data given in Table 2.

### 3.3. Interactions of Endonuclease III with F-Ligand

**Figure 3:**
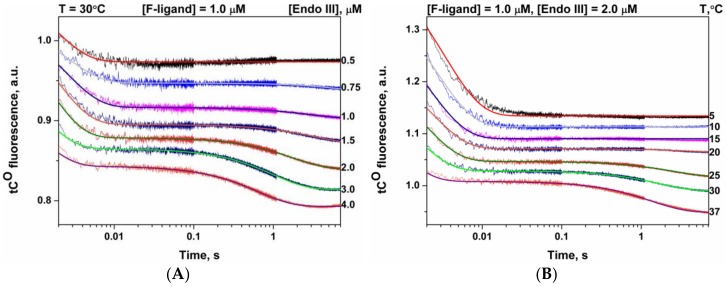
(**A**) Changes in tC^O^ fluorescence intensity during the interaction of Endo III with F-ligand (1 µM) at various concentrations of the enzyme at 30 °C. Solid curves represent the curves fitted by means of Scheme 2; (**B**) Changes in tC^O^ fluorescence intensity during the interaction of Endo III (2 µM) with F-ligand (1 µM) at different temperatures.

**Scheme 2:**
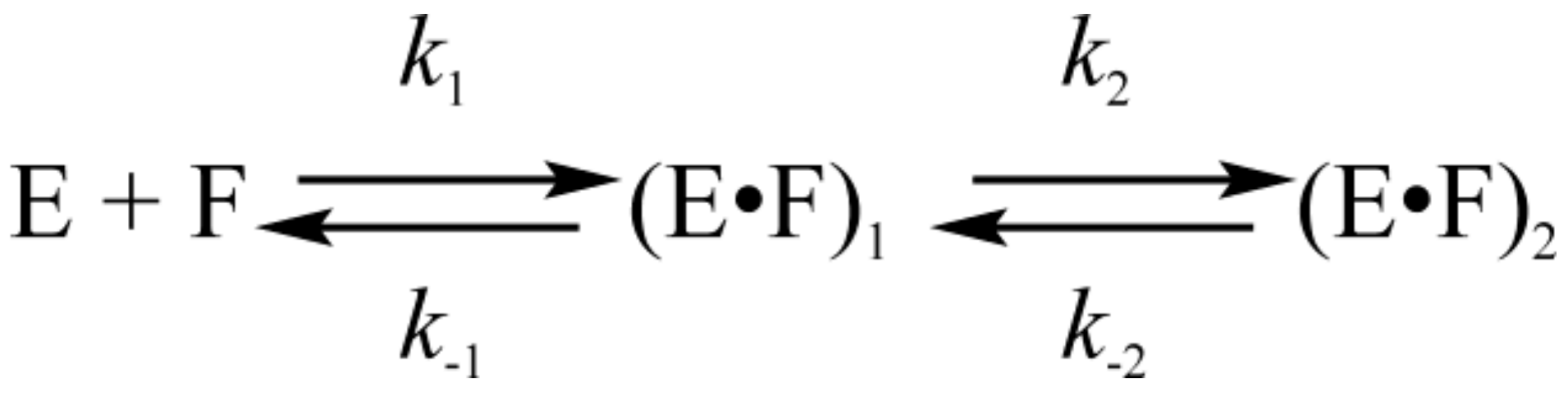
E represents Endo III, F denotes F-ligand, (E•F)_i_ are complexes of the enzyme with F-ligand, and *k*_i_ and *k*_−i_ are the individual rate constants.

Binding of F-ligand by Endo III induces two-phase changes in tC^O^ fluorescence (Figure 3A). The phase of a fast fluorescence intensity decrease was completed by 10 ms and followed by the second decrease phase up to 5–10 s, which was observed only at T = 20–37 °C. Therefore, the stopped-flow data obtained for a set of Endo III concentrations and a fixed DNA concentration at each temperature (Figure 3B) were fitted to an equilibrium mechanism involving two reversible steps, as shown in Scheme 2, and the forward and reverse rate constants were calculated. The rate and equilibrium constants are presented in Table 3.

### 3.4. Interactions of Endonuclease III with DHU-Substrate

**Figure 4:**
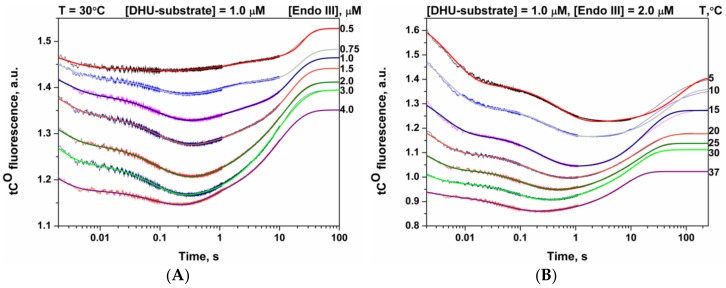
(**A**) Changes in tC^O^ fluorescence intensity during the interaction of Endo III with DHU-substrate (1 µM) at various concentrations of the enzyme at 30 °C. Solid lines represent the curves fitted by means of Scheme 3; (**B**) Changes in tC^O^ fluorescence intensity during the interaction of Endo III (2 µM) with DHU-substrate (1 µM) at different temperatures.

For DHU-substrate, the set of tC^O^ fluorescence traces obtained at different concentrations of Endo III and T = 30 °C, are illustrated in Figure 4A. As shown in Figure 4A, the tC^O^ fluorescence curve contains at least four phases: two initial phases of an intensity decrease (up to 10 ms and 200 ms, respectively) and two phases of the intensity increase (up to 5 s and 30 s). Figure 4B illustrates the fluorescence traces obtained for 2 µM Endo III and 1 µM DHU-containing DNA substrate at different temperatures.

**Scheme 3:**
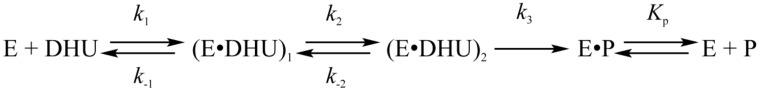
E represents Endo III, DHU denotes DHU-substrate, (E•DHU)_i_ are the enzyme–substrate complexes, E•P is the enzyme–product complex, and *k*_i_ and *k*_−i_ are the individual rate constants.

For each temperature, the set of fluorescence traces obtained at different concentrations of Endo III (0.5–4.0 μM) was fitted, as described in our previous study [19], to a kinetic mechanism (Scheme 3) containing two reversible steps, representing the sequential recognition of the damaged site and formation of the enzyme’s catalytically competent conformation. The rate constants of the elementary steps estimated based on this kinetic scheme are listed in Table 4.

**Figure 5:**
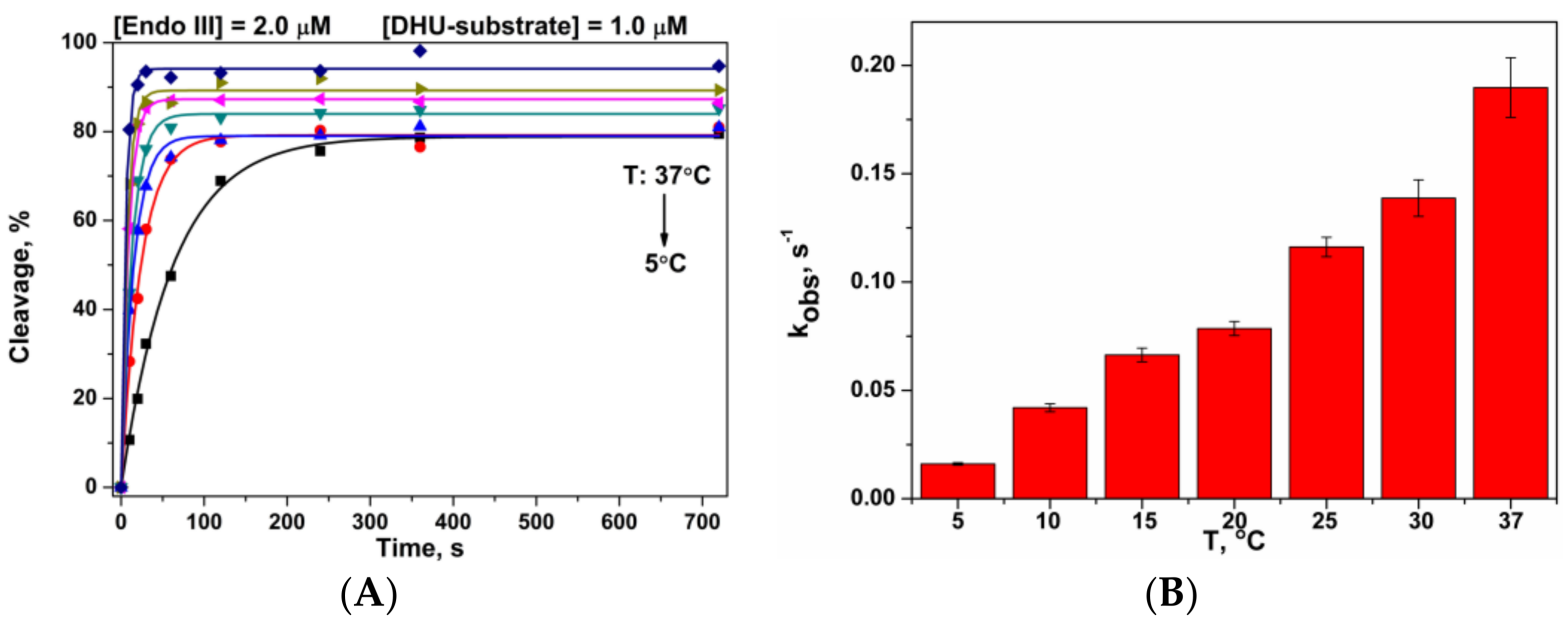
(**A**) Kinetics of accumulation of the DNA product formed in the *N*-glycosylase and apurinic/apyrimidinic (AP)-lyase reactions, as detected by the polyacrylamide gel electrophoresis (PAGE) experiments. To visualize kinetic curves in the initial time region different colors are used. (**B**) The observed rate constants for DHU-substrates’ cleavage by Endo III at different temperatures, [Endo III] = 2 μM, [DHU-substrate] = 1 μΜ.

As follows from the PAGE analysis of the product accumulation presented in Figure 5, the third phase of tC^O^ fluorescence corresponds to the catalytic step of the process, which includes both the *N*-glycosylase and AP-lyase reactions. It was shown previously [19] the AP lyase activity does not limit the reaction and proceeds with a rate similar to the rate of the *N*-glycosylase reaction. This is consistent with the observation of only a single fluorescence change in DHU-substrate cleavage (Figure 4) and makes it impossible to separate the chemical stages of the process into separate steps of the kinetic mechanism (Scheme 3). The characteristic times of the DNA product accumulation coincide with the characteristic times of tC^O^ fluorescence increase in the 1–100 s interval, indicating that the fluorescence changes in this time interval characterize the chemical steps of the enzymatic reaction. The third phase shifts to a shorter timescale with an increase in Endo III concentration or temperature (Figure 4A,B, respectively). Therefore, the first two phases correspond to the binding of the enzyme to the substrate and formation of a catalytically competent enzyme–substrate complex. In Figure 4A,B, the fluorescence intensities of the enzyme–DNA complexes (0.002–0.3 s) are lower in comparison with the free double-stranded DNA (the initial part of the traces, short timescales) and with the final enzyme–DNA product complex formed after removal of the DHU base and β-elimination of the 3′-phosphate group (the ending of the traces, long timescales).

Of note, the first decrease phase, which corresponds to nonspecific-complex formation when the enzyme distorts a DNA chain and the fluorophore environment becomes more polar, has similar shapes for DHU-substrate and G- and F-ligands. Nevertheless, the association constant of this step *K*_1_ increases in the order G-ligand → F-ligand → DHU-substrate (0.17, 0.52, and 0.8 μM^−1^ at 25°C) indicating stabilization of the initial complex for damaged DNA.

The second step of the reduction in tC^O^ fluorescence intensity is associated with recognition of the DHU base and its eversion from the DNA helix, as was suggested earlier [19]. In this case, tC^O^ becomes more accessible for polar molecules of water. At this step, a significant difference between specific DHU-substrate (a tC^O^ fluorescence decrease) and nonspecific G-ligand (a tC^O^ fluorescence increase) was detected, indicating that the second step is the most important for discrimination between damaged and undamaged DNA.

The third phase, when the fluorescence signal is increasing in the case of DHU-substrate, corresponds to transformation of the enzyme–substrate complex into the catalytically active conformation suitable for subsequent *N*-glycosylase and AP-lyase reactions. The X-ray data obtained for Endo III from *G. stearothermophilus* revealed that, in the complex of Endo III with DHU-substrate, amino acid residues Leu82 and Gln42 are inserted into the DNA helix (Leu81 and Gln41 in Endo III from *E. coli*) and 5,6-dihydrouracil is everted into the active site pocket (Figure 1). These structural transformations make the tC^O^ environment less polar and cause a gain of the fluorescence signal. The final fourth phase when the signal reaches a plateau corresponds to reversible dissociation of the enzyme–product complex.

### 3.5. Thermodynamic Analysis

**Figure 6:**
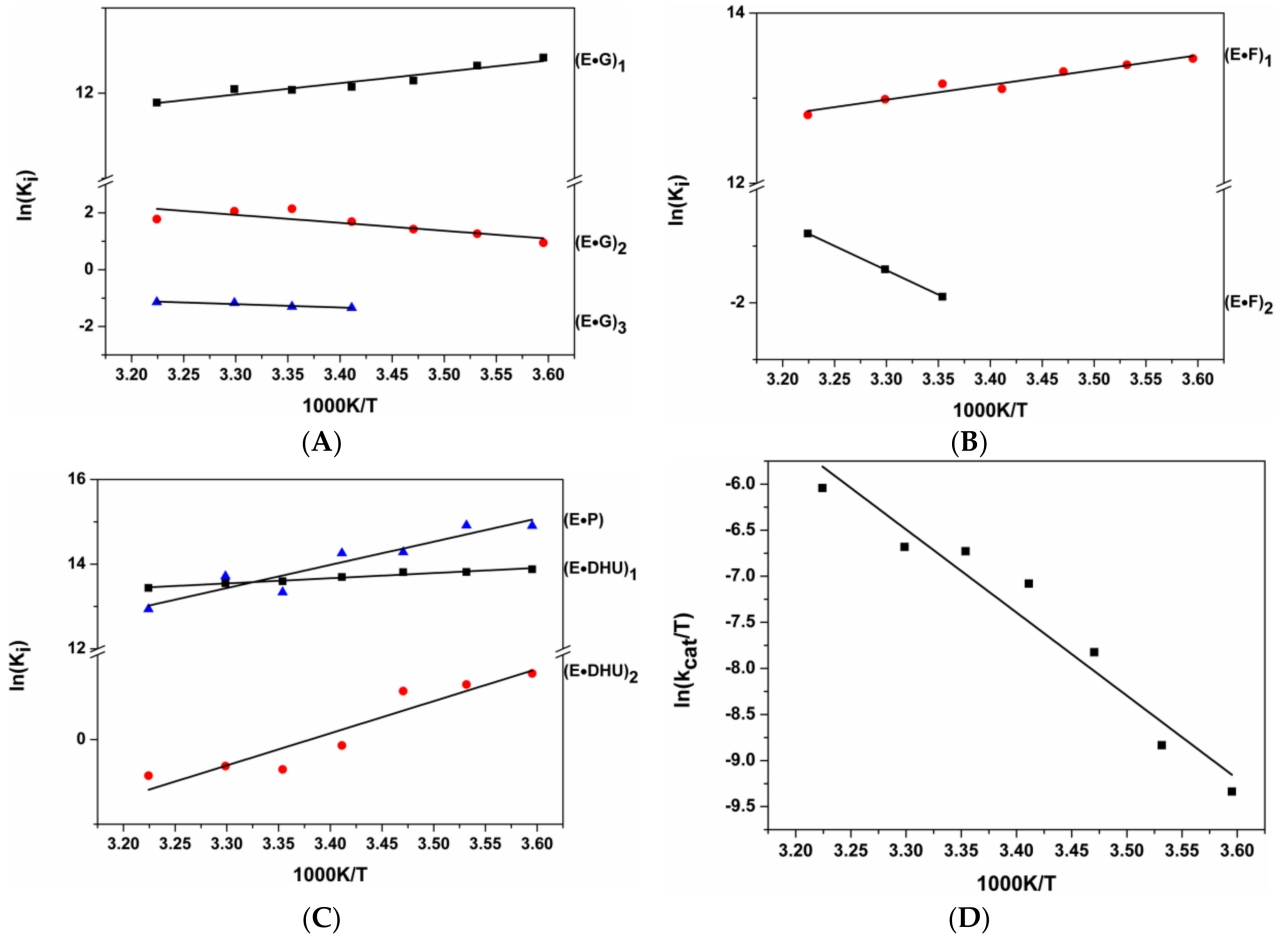
Van’t Hoff analysis of the temperature dependence of *K*_i_ for: (**A**) G-ligand; (**B**) F-ligand; and (**C**) DHU-substrate; (**D**) Analysis of the temperature dependence of ln(*k*_cat_/T) for DHU-substrate.

From the values of individual rate constants, equilibrium constants *K*_i_ (*k*_i_/*k*_−i_, where *i* is the step number) were computed for G- and F-ligands and DHU-substrate. Δ*H*^o^_i_ and Δ*S*^o^_i_ were calculated using the Van’t Hoff equation: ln(*K*_i_) = Δ*S*^o^_i_/R − Δ*H*^o^_i_/RT. It should be noted that the slope of the plot ln(*K*_i_) vs. 1/RT is equal to the standard enthalpy of the reaction irrespective of the assumption of temperature independence of standard enthalpy, as follows from the Gibbs–Helmholtz equation [46]. As depicted in Figure 6, the dependences ln(*K*_i_) vs. 1/T are linear, as expected for the relatively narrow temperature range of the study. The Gibbs free energies Δ*G*^o^_i_ at 25 ^°^C (298 K) were calculated according to ln(*K*_i_) = −Δ*G*^o^_i_/RT (Table 5).

Inspection of the thermodynamic data summarized in Table 5 reveals that formation of the first complex of Endo III with any DNA is associated with a negative change in enthalpy and a positive change in entropy, indicating similar nature of the interactions in the initial enzyme–DNA complex. Nevertheless, these data revealed a clear qualitative difference in the total thermodynamic changes between the processes of Endo III binding to nonspecific and specific DNA substrates. The total binding of G- and F-ligands is associated with positive changes in enthalpy and entropy, but the binding of specific DHU-substrate is fully enthalpy-driven due to the exothermic second binding step. Indeed, the second step of DHU-substrate binding reflected adjustment of the enzyme–substrate complex into the catalytically active state and is characterized by negative changes in both enthalpy and entropy. The negative Δ*H*°_2_ value indicates stabilization of the complex during formation of new, energetically favorable bonds among the interacting atoms, whereas a negative Δ*S*°_2_ value points to compaction and increased rigidity of the complex.

Therefore, discrimination between a nonspecific G base and the specific DHU base occurs at the second binding step, which manifests the significant difference in the thermodynamic parameters, indicating the difference in the nature of interactions during this step.

### 3.6. Mutational Analysis

**Figure 7:**
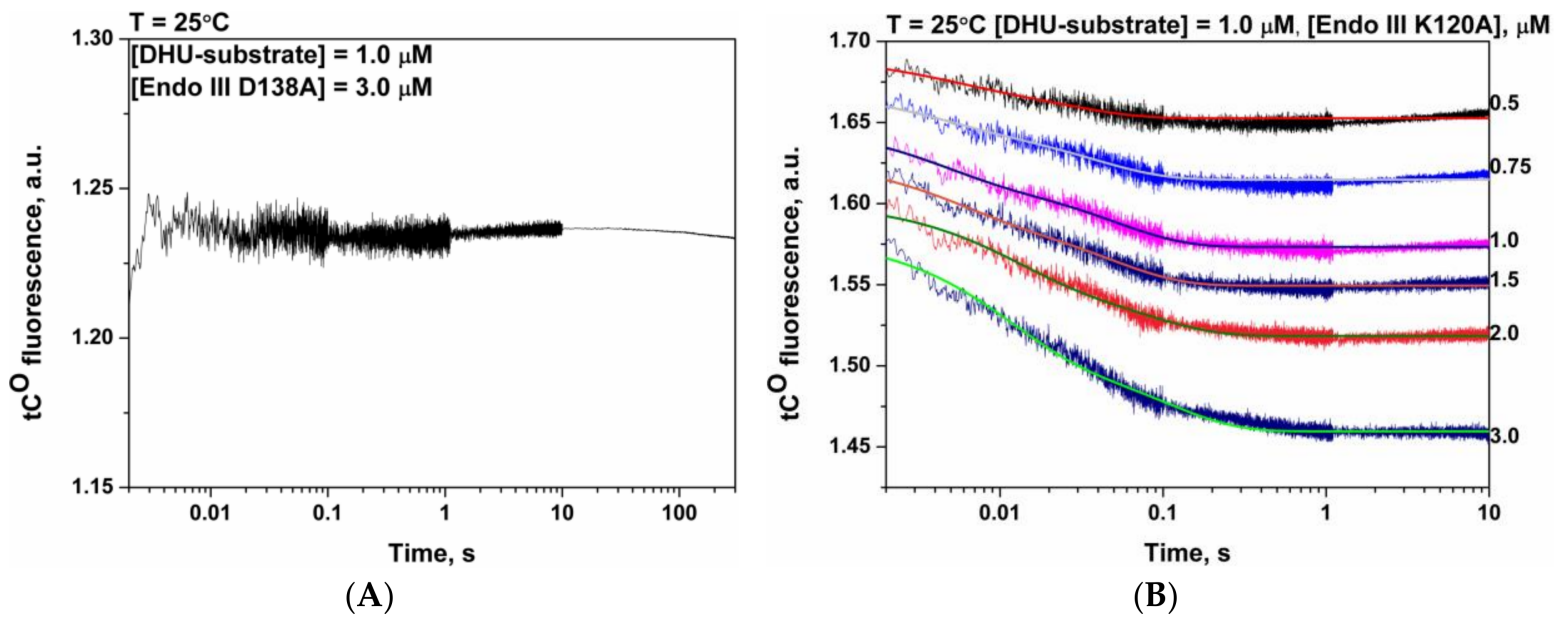
Changes in tC^O^ fluorescence intensity during the interaction of Endo III D138A (**A**) or Endo III K120A (**B**) with DHU-substrate (1 µM) at 25 °C. Solid curves represent the curves fitted via the binding steps of Scheme 3.

To identify the role of Lys120 and Asp138 in the hydrolysis of the *N*-glycosidic bond and in β-elimination of the 3′-phosphate group, mutant forms Endo III K120A and Endo III D138A were constructed. Analysis of their catalytic activity via detection of the reaction products by gel electrophoresis showed that substitution of the catalytic amino acid residues (Lys120Ala and Asp138Ala) results in a complete loss of both the *N*-glycosylase and AP-lyase activities. Analysis of DNA conformational changes induced by the mutant forms revealed that substitution D138A leads to a complete loss of the ability to distort the DNA double chain, probably due to loss of the ability to tightly DNA binding (Figure 7A).

The decrease in tC^O^ fluorescence during Endo III K120A interaction with DHU-substrate indicates a similar nature of the DNA-binding steps recorded in initial parts of the kinetic curves for the WT enzyme (Figure 7B). On the other hand, the shapes of kinetic curves revealed significant deceleration of the binding process in comparison with the WT enzyme. It was not possible to detect an increase in fluorescence intensity, which characterizes accumulation of the reaction product and dissociation of the enzyme–product complex because of the extremely slow rate of this step. Therefore, the kinetic curves were described by two reversible binding steps of Scheme 3, which allowed us to calculate the rate and equilibrium constants for the binding of Endo III K120A to DHU-substrate (Table 6).

As shown in Table 6, in the case of Endo III K120A, *k*_1_ and *k*_2_ are less than those for WT Endo III (at 25 °C) by 14.5- and 2.4-fold, respectively. The association constant of the first binding step obtained for Endo III K120A is also six-fold lower as compared with the WT enzyme, indicating participation of Lys120 in the initial binding step. The second step—where the insertion of amino acid residue Gln41 into the DNA double helix takes place (and the catalytic complex should be formed)—proceeds with similar forward rate constants in the case of both WT Endo III and K120A mutant. Nonetheless, the comparison of the equilibrium constants presented in Table 4 and Table 6 reveals a decrease in *K*_2_ and in total *K*_ass_ for Endo III K120A by 1.8- and 8.3-fold.

These results suggest that the conformation of the active site of Endo III K120A is less suitable for the specific DNA binding, and this process proceeds less effectively than in the case of the WT enzyme. Moreover, for the K120A mutant form, full adaptation of the DNA helix structure to the catalytically active form is not possible.

## 4. Discussion

Crystal structures of free Endo III from *E. coli* [17,49] and complexes of Endo III from *G. stearothermophilus* with damaged DNA [18] revealed a significant distortion of the DNA double chain at the site of the lesion, with the DNA axis kinked by ≈55°, the minor groove widely open, and the damaged nucleotide everted from the helix into the enzyme’s active site pocket.

The fluorescent reporter, tC^O^, incorporated opposite to the lesion enables recording a two-phase decrease in its fluorescence during the F-ligand and DHU-substrate binding by Endo III (Figure 3 and Figure 4). On the contrary, binding of G-ligand leads to an initial single-phase decrease with a subsequent increase phase and second decrease phase (Figure 2).

It has been uncovered earlier that the fluorescence quantum yield of tC^O^ decreases after switching to a more polar environment [48], when it was still 0.22 ± 0.05 in all possible combinations of nearest neighbors [50]. According to thermodynamic data (Table 5), the first step is characterized by similar thermodynamic parameters for all DNA duplexes containing residues G, F, and DHU. At this step, the moderate enthalpy gain is accompanied by an increase in entropy most probably due to the DNA melting at the point of contact and conformational changes of the DNA-binding site. The Gibbs free energy of binding (ca. −7 kcal/mol) is typical for protein–DNA complexes [51]. Formation of the protein–DNA complex restrains translational, rotational, and conformational motions of the protein and DNA, thereby leading to the entropy loss. The entropy gain is provided by a release of water molecules from the protein–DNA interface, which has been reported to accompany molecular complexation in many systems, including protein–DNA complexes. As illustrated in Figure 2, Figure 3 and Figure 4, disruption of DNA base pairing proceeds after formation of the initial contacts with Endo III. Such disturbance of the DNA conformation in both specific and nonspecific complexes may result from the attempts of the enzyme to flip out the sampled base regardless of whether it is damaged. Active eversion of undamaged bases from DNA in the process of a lesion search has also been demonstrated for hOGG1 [52,53]. Besides, in all available structures of DNA glycosylases with undamaged DNA [54,55,56,57], the wedging residue of the enzyme is already inserted into DNA, and therefore Leu81 of Endo III may be expected to behave in the same way as shown recently in single-molecule assays in Refs. [23,58,59,60]. Therefore, the possible molecular event causing a tC^O^ fluorescence decrease in the first phase of nonspecific DNA binding may be the insertion of Leu81 as part of the lesion search, affecting the environment of tC^O^ in fully stacked undamaged and damaged DNA.

The second phase of interaction with DHU-substrate and F-ligand (Figure 2, Figure 3 and Figure 4) also leads to a decrease in the tC^O^ fluorescence signal, which reflects formation of the catalytically competent state. Nevertheless, thermodynamic parameters of this step are significantly different. The second step for DHU-substrate has favorable enthalpy but unfavorable entropy. At this step, the damaged nucleotide is pulled into the enzyme’s active site pocket, and new contacts between amino acids of the active site and the DHU base as well as between the amino acids Leu81 and Gln41 of Endo III with DNA in the voids are formed, thereby leading to the energy gain. By contrast, the resulting structure is more rigid, which explains the entropy loss. In the case of F-ligand, the absence of a damaged base results in the loss of these specific contacts, thus causing a positive change in enthalpy and entropy at this step. The consumption of energy (Δ*H*°) at this step is compensated by the gain of entropy (Δ*S*°), presumably due to the dehydration of the DNA grooves. As a result, this step is neutral in terms of Gibbs energy for both DHU-substrate and F-ligand; this pattern is very important for transformation of the structure of the enzyme–DNA complex to the catalytically active conformation. 

In the case of nonspecific G-ligand during the second phase, the enzyme forms specific contacts with both chains of DNA. As suggested previously [19], during the DNA binding—except for the side chain of Leu81 of Endo III (which can be partially inserted into the DNA duplex during complex formation)—the side chain carbonyl of Gln41 of Endo III contacts the base opposite to the lesion site. Therefore, due to these interactions, the tC^O^ environment becomes less polar and the tC^O^ fluorescence intensity increases. In the third phase, tC^O^ fluorescence intensity decreases again. Probably, the insertion of amino acid residues into the double helix of DNA promotes base pair opening and extrusion of nucleobases including tC^O^ into the external polar side of the DNA chain. The third step for G-ligand is energetically neutral in terms of Gibbs energy but proceeds very slowly. This step likely reflects an attempt of the enzyme to evert any base of nonspecific DNA into the enzyme’s active site. 

The third step of the Endo III interaction with DHU-substrate represents a catalytic *N*-glycosylase reaction and a reaction of β-elimination of the 3′-phosphate group. Activation parameters of this reaction reveal a sufficiently low value of Δ*G*^≠^, explaining the high activity of the enzyme at low temperatures. 

The fourth step for DHU-substrate is the equilibrium between the complex of Endo III with the product and their free forms in solution.

## 5. Conclusions

The thermodynamic analyses of catalytic processes performed by functionally and structurally different DNA-glycosylases Endo III from *E. coli*, Fpg from *E. coli* [31], human OGG1 [30] and human AP-endonuclease APE1 [32] reveal that they employ common energetic features at the main steps of base lesion recognition. The first step of the DNA-binding process is formation of a nonspecific complex, resulting in double-helix melting and insertion of wedging amino acid residues (Leu81 for Endo III, Phe110 for Fpg and Tyr203 for hOGG1), characterized by favorable changes in both enthalpy and entropy for all enzymes except APE1. Common feature of Endo III, Fpg and APE1 is that some of the subsequent steps in a multistep mechanism of lesion recognition are favorable in terms of enthalpy but may be unfavorable on entropy owing to the formation of structurally rigid complexes. Moreover, at least one step of DNA binding by any enzyme required additional energy consumption, which is compensated by an increase in entropy of the system as a result of release of water molecules from the area of contacts.

Mutational analysis of Endo III reveals that Lys120 takes part both in the processes of nonspecific binding and subsequent recognition of the damage because substitution Lys120Ala significantly decelerates conformational changes of the duplex during the complex formation. Moreover, substitution Asp138Ala causes a complete loss of the ability of Endo III to distort a DNA double chain.

## Figures and Tables

**Table 1:** DNA duplexes used as substrates and ligands of Endo III. DHU: 5,6-dihydrouracil; F: (3-hydroxytetrahydrofuran-2-yl)methyl phosphate.

Shorthand	Sequence
DHU-substrate, X = DHU F-ligand, X = F-site G-ligand, X = G	5′-TCTCTCTC X CCTTCCTT-3′ 3′-AGAGAGAG(tC^O^)GGAAGGAA-5′

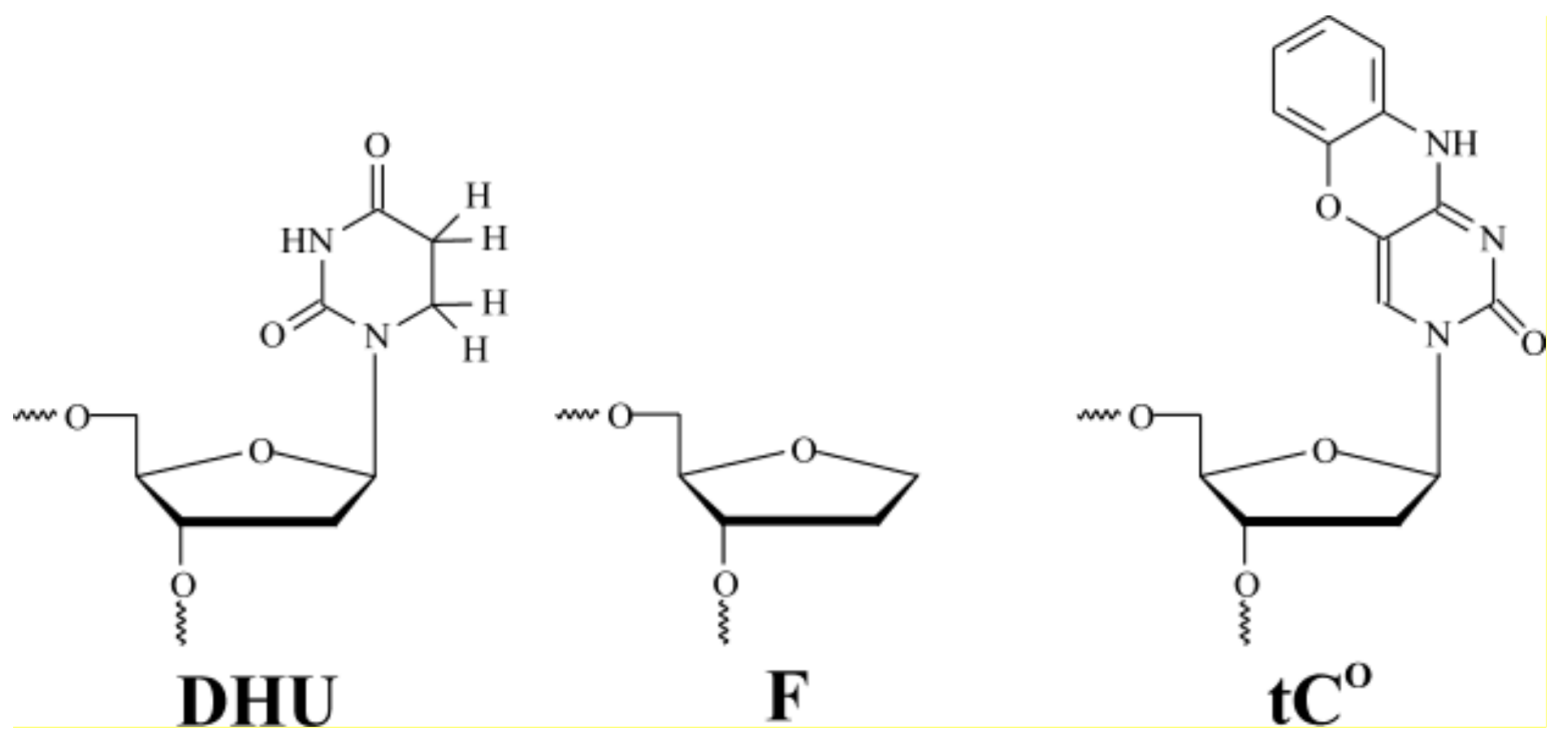

**Table 2:** Rate and equilibrium constants corresponding to the interaction of Endo III with G-ligand.

T (°C) *Constants*	5	10	15	20	25	30	37
*k*_1_, M^−1^ s^−1^	(27 ± 2)×10^6^	(30 ± 4)×10^6^	(45 ± 5)×10^6^	(52 ± 5)×10^6^	(61 ± 4)×10^6^	(65 ± 11)×10^6^	(72 ± 3)×10^6^
*k*_−1_, s^−1^	110 ± 3	131 ± 11	239 ± 11	299 ± 20	360 ± 14	379 ± 36	494 ± 24
*K*_1_, M^−1^	(0.25 ± 0.01)×10^6^	(0.23 ± 0.04)×10^6^	(0.19 ± 0.02)×10^6^	(0.18 ± 0.02)×10^6^	(0.17 ± 0.01)×10^6^	(0.17 ± 0.03)×10^6^	(0.15 ± 0.01)×10^6^
*k*_2_, s^−1^	0.034 ± 0.006	0.15 ± 0.05	0.33 ± 0.01	0.47 ± 0.14	0.58 ± 0.14	0.9 ± 0.3	1.2 ± 0.1
*k*_−2_, s^−1^	0.013 ± 0.003	0.04 ± 0.01	0.078 ± 0.004	0.090 ± 0.030	0.070 ± 0.030	0.120 ± 0.060	0.200 ± 0.050
*K*_2_	2.6 ± 0.8	3.5 ± 1.8	4.2 ± 0.2	5.42 ± 2.55	8.5 ± 4.2	7.8 ± 4.5	5.9 ± 1.5
*k*_3_, s^−-1^	N/A	N/A	N/A	0.004 ± 0.002	0.014 ± 0.004	0.017 ± 0.003	0.029 ± 0.006
*k*_−3_, s^−1^	N/A	N/A	N/A	0.015 ± 0.003	0.050 ± 0.004	0.054 ± 0.016	0.09 ± 0.04
*K*_3_	N/A	N/A	N/A	0.26 ± 0.12	0.27 ± 0.09	0.3 ± 0.1	0.32 ± 0.15
*K*_ass_, M^–1^	(0.9 ± 0.01)×10^6^	(1.04 ± 0.04)×10^6^	(0.99 ± 0.02)×10^6^	(1.4 ± 0.02)×10^6^	(2.0 ± 0.01)×10^6^	(1.9 ± 0.03)×10^6^	(1.3 ± 0.01)×10^6^

*K_i_ = k_i_/k_−i_, K_ass_* = $\sum_{i=1}^{i=3}\prod_{j=1}^{j=i}K_{j}$

**Table 3:** Rate and equilibrium constants corresponding to the interaction of Endo III with F-ligand.

T (°C) Constants	5	10	15	20	25	30	37
*k*_1_, M^−1^ s^−1^	(86 ± 15)×10^6^	(98 ± 17)×10^6^	(102 ± 20)×10^6^	(106 ± 10)×10^6^	(156 ± 15)×10^6^	(172 ± 6)×10^6^	(192 ± 27)×10^6^
*k*_−1_, s^−1^	122 ± 12	150 ± 18	169 ± 15	214 ± 15	298 ± 50	395 ± 6	528 ± 34
*K*_1_, M^−1^	(0.71 ± 0.14)×10^6^	(0.65 ± 0.14)×10^6^	(0.60 ± 0.13)×10^6^	(0.49 ± 0.06)×10^6^	(0.52 ± 0.1)×10^6^	(0.44 ± 0.017)×10^6^	(0.36 ± 0.06)×10^6^
*k*_2_, s^−1^	N/A	N/A	N/A	0.005 ± 0.001	0.06 ± 0.01	0.19 ± 0.01	0.23 ± 0.06
*k*_−2_, s^−1^	N/A	N/A	N/A	0.17 ± 0.04	0.37 ± 0.02	0.804 ± 0.003	0.50 ± 0.02
*K*_2_	N/A	N/A	N/A	0.03 ± 0.01	0.15 ± 0.04	0.24 ± 0.01	0.46 ± 0.1
*K*_ass_, M^−1^	(0.71 ± 0.14)×10^6^	(0.65 ± 0.14)×10^6^	(0.60 ± 0.13)×10^6^	(0.50 ± 0.06)×10^6^	(0.41 ± 0.10)×10^6^	(0.54 ± 0.17)×10^6^	(0.52 ± 0.06)×10^6^

*K*_i_ = *k*_i_*/k*_−i_, *K*_ass_ = $\sum_{i=1}^{i=2}\prod_{j=1}^{j=i}K_{j}$.

**Table 4:** Rate and equilibrium constants corresponding to the interaction of Endo III with DHU-substrate.

T (°C) *Constants*	5	10	15	20	25	30	37
*k*_1_, M^−1^ s^−1^	(50 ± 7)×10^6^	(63 ± 11)×10^6^	(88 ± 8)×10^6^	(98 ± 16)×10^6^	(116 ± 5)×10^6^	(127 ± 30)×10^6^	(144 ± 7)×10^6^
*k*_−1_, s^−1^	47 ± 6	63 ± 11	88 ± 15	111 ± 17	145 ± 11	166 ± 16	210 ± 35
*K*_1_, M^−1^	(1.1 ± 0.2)×10^6^	(1.0 ± 0.3)×10^6^	(1.0 ± 0.2)×10^6^	(0.9 ± 0.2)×10^6^	(0.8 ± 0.1)×10^6^	(0.8 ± 0.2)×10^6^	(0.7 ± 0.1)×10^6^
*k*_2_, s^−1^	2.1 ± 0.6	2.6 ± 0.4	3.5 ± 0.3	3.8 ± 0.4	3.8 ± 0.4	5.3 ± 1.2	9.5 ± 1.5
*k*_−2_, s^−1^	1.0 ± 0.1	1.3 ± 0.1	2.0 ± 0.4	4.1 ± 0.5	5.5 ± 0.2	7.2 ± 1.3	14.5 ± 1.2
*K*_2_	2.2 ± 0.7	1.9 ± 0.4	1.8 ± 0.4	0.9 ± 0.1	0.7 ± 0.1	0.7 ± 0.2	0.65 ± 0.12
*k*_3_, s^−1^	0.027 ± 0.006	0.045 ± 0.007	0.13 ± 0.03	0.27 ± 0.06	0.39 ± 0.04	0.41 ± 0.09	0.8 ± 0.2
*K*_P_, M^−1^	(2.9 ± 1.7)×10^6^	(3.0 ± 2)×10^6^	(1.6 ± 1.4)×10^6^	(1.6 ± 1.4)×10^6^	(0.62 ± 0.5)×10^6^	(0.9 ± 0.4)×10^6^	(0.4 ± 0.1)×10^6^
*K*_ass_, M^–1^	(3.5 ± 0.2)×10^6^	(2.9 ± 0.3)×10^6^	(2.8 ± 0.2)×10^6^	(1.7 ± 0.2)×10^6^	(1.5 ± 0.1)×10^6^	(1.4 ± 0.2)×10^6^	(1.2 ± 0.1)×10^6^

*K*_i_ = *k*_i_*/k*_-i_, *K*_ass_ = $\sum_{i=1}^{i=2}\prod_{j=1}^{j=i}K_{j}$.

**Table 5:** Thermodynamic parameters of Endo III interactions with DNA.

DNA	Step Number	Δ*H*, kcal/mol	Δ*S*, cal/(mol × K)	Δ*G*_298_, kcal/mol ^a^
G-ligand	1	−2.6 ± 0.3	15.1 ± 1.1	−7.1
	2	5.5 ± 1.5	22 ± 5	−1.3
	3	2.3 ± 0.5	5.3 ± 1.7	0.7
	$\sum_{i=1}^{i=3}$	5.2 ± 2.3	42.4 ± 7.8	−7.7
F-ligand	1	−3.5 ± 0.4	14 ± 1	−7.8
	2	17.0 ± 0.1	53.3 ± 0.5	1.1
	$\sum_{i=1}^{i=2}$	13.5 ± 0.5	67.3 ± 1.5	−6.7
DHU-substrate	1	−2.4 ± 0.2	19 ± 1	−8.0
	2	−7.5 ± 1.2	−25 ± 4	0.2
	$\sum_{i=1}^{i=2}$	−9.9 ± 1.4	−6.0 ± 5.0	−7.8
	Transition state of catalytic step 3 ^b^	18 ± 2	−1.0 ± 0.1	18.0
	Product dissociation step 4	−11 ± 2	9 ± 6	−7.9

^a ^The errors indicated are ± 1 standard deviation. ∆∆*G*°_i298_ = RT(∆*K*_i_/*K*_i_) ≤ 0.2 kcal/mol. ^b ^Apparent thermodynamic parameters of the transition state calculated as ∆*G_3_*^‡^ = RT ln(*k*_B_T/*hk*_3_), where *k*_B_ and *h *are Boltzmann and Planck constants, respectively, R is the gas constant, and T is absolute temperature in Kelvins.

**Table 6:** Rate and equilibrium constants corresponding to the interaction of Endo III K120A with DHU-substrate.

Constants	T (25 °C)
*k*_1_, M^−1^ s^−1^	(8 ± 2) × 10^6^
*k*_−1_, s^−1^	60 ± 15
*K*_1_, M^−1^	(0.13 ± 0.05) × 10^6^
*k*_2_, s^−1^	4.6 ± 1.4
*k*_−2_, s^−1^	12 ± 2
*K*_2_	0.4 ± 0.1
*K*_ass_, M^–1^	0.18 × 10^6^

*K*_i_ = *k*_i_*/k*_−i_, *K*_ass_ = $\sum_{i=1}^{i=2}\prod_{j=1}^{j=i}K_{j}$.
